# Nrf2 gene mutation and single nucleotide polymorphism rs6721961 of the Nrf2 promoter region in renal cell cancer

**DOI:** 10.1186/s12885-019-6347-0

**Published:** 2019-11-21

**Authors:** Yoshiyuki Yamaguchi, Takao Kamai, Satoru Higashi, Satoshi Murakami, Kyoko Arai, Hiromichi Shirataki, Ken-Ichiro Yoshida

**Affiliations:** 1 Department of Urology, Dokkyo Medical University, 880 Kitakobayashi Mibu, Tochigi, 321-0293 Japan; 2 Department of Molecular and Cell Biology, Dokkyo Medical University, Mibu, Tochigi, Japan; 3 Division of Field Application, Life Technologies, Tokyo, Japan

**Keywords:** Nuclear factor erythroid 2–related factor 2 (Nrf2), Kelch-like ECH-associated protein 1 (Keap1), Single nucleotide polymorphism, Renal cell carcinoma

## Abstract

**Background:**

Nuclear factor erythroid 2–related factor 2 (Nrf2) is involved in cell proliferation by promotion of metabolic activity. It is also the major regulator of antioxidants and has a pivotal role in tumor cell proliferation and resistance to chemotherapy. Accordingly, we investigated the role of Nrf2 in renal cell carcinoma (RCC).

**Methods:**

In 50 patients who had metastatic RCC and received cytoreductive nephrectomy, we performed Nrf2 gene mutation analysis using targeted next-generation sequencing, as well as investigating a specific single nucleotide polymorphism (SNP; rs6721961) in the Nrf2 promoter region and Nrf2 protein expression.

**Results:**

Targeted next-generation sequencing revealed that five tumors had SNPs of Nrf2 associated with amino acid sequence variation, while 11 tumors had SNPs of Kelch-like ECH-associated protein 1 gene, 35 had SNPs of von Hippel-Lindau gene, and none had SNPs of fumarate hydratase gene. The three genotypes of rs6721961 showed the following frequencies: 60% for C/C, 34% for C/A, and 6% for A/A. Nrf2 mutation and the C/A or A/A genotypes were significantly associated with increased Nrf2 protein expression (*p* = 0.0184 and *p* = 0.0005, respectively). When the primary tumor showed Nrf2 gene mutation, the C/A or A/A genotype, or elevated Nrf2 protein expression, the response of metastases to vascular endothelial growth factor-targeting therapy was significantly worse (*p* = 0.0142, *p* = 0.0018, and *p* <  0.0001, respectively), and overall survival was significantly reduced (*p* = 0.0343, *p* = 0.0421, and *p* <  0.0001, respectively). Elevated Nrf2 protein expression was also associated with shorter survival according to multivariate Cox proportional analysis.

**Conclusion:**

These findings suggest an associated between progression of RCC and Nrf2 signaling.

## Background

Activation of nuclear factor erythroid-2-related factor 2 (Nrf2) increases tumor cell resistance to chemotherapy and promotes growth, so there is an association between elevated tumor expression of Nrf2 protein and a poor prognosis [1–3]. Constitutive activation of Nrf2 was reported to increase metabolic activity and cell proliferation [4], which may be important for development and progression of cancer. However, the Kelch-like ECH-associated protein 1 (Keap1) - Nrf2 pathway is the major regulator of protective cellular responses to both oxidative and electrophilic stress, which means that Nrf2 acts to prevent carcinogenesis in normal or premalignant tissues. Thus, Nrf2 is a classic double-edged sword that can prevent or promote cancer, depending on the cellular context and microenvironment [5].

Somatic mutations of Nrf2 have been reported in many human cancers, with tumorigenic mutations typically leading to activation of Nrf2 targets [1–3]. Nrf2 gene mutations have been reported to lead to modification of certain residues in Nrf2 protein [6]. In addition, Nrf2 promoter alterations like single nucleotide polymorphisms (SNPs) were reported to cause marked repression of both Nrf2 transcription and activity [6]. NRF2 binds to antioxidant response elements (ARE) and up-regulates protective detoxifying enzymes in response to oxidative stress. Multiple SNPs of the Nrf2 gene have been identified [7, 8]. For example, Shimoyama et al. reported the association of a SNP (rs35652124) with cardiovascular mortality in hemodialysis patients [9]. In addition, a SNP (rs6721961) in the promoter region of Nrf2 (Nrf2 regulatory SNP-617) was reported to be involved in carcinogenesis [7, 8], and is associated with a significantly higher risk of developing non-small cell lung cancer [10]. It has also been reported that this SNP (rs6721961) is associated with a higher risk of several cardiovascular diseases, including venous thromboembolism [11], reduced vital capacity [12], and an impaired forearm vasodilator response [13]. Thus, SNP (rs6721961) in the promoter region of Nrf2 (Nrf2 regulatory SNP (rSNP)-617) seems to influence Nrf2 expression [10]. Research on the role of Nrf2 in human renal cell carcinoma (RCC) has been mainly focused on papillary type 2 RCC (pRCC2), since an aggressive form of this tumor is characterized by increased oxidative stress and activation of the Nrf2-ARE pathway [14, 15]. It was recently reported that the Nrf2 pathway is also associated with progression of clear cell RCC (ccRCC) [16–18], but the role of Nrf2 in ccRCC has not been fully investigated. In order to shed more light on the influence of Nrf2 signaling in human ccRCC, we assessed Nrf2 gene mutations, the rs6721961 SNP, and Nrf2 protein expression in patients with metastatic ccRCC, as well as associations with the response to adjuvant vascular endothelial growth factor (VEGF)-targeting therapy and survival.

## Methods

### Patients

We retrospectively investigated 50 patients (33 men and 17 women) who had a confirmed histopathological diagnosis of metastatic clear cell RCC (ccRCC) and received cytoreductive nephrectomy at our hospital from 2012 to 2017. Nephrectomy was done before they received other treatment. Preoperative CT and/or MRI was performed in all patients for tumor staging. The median postoperative follow-up period was 27 months (range: 4–72 months).

Following cytoreductive nephrectomy, first-line adjuvant vascular endothelial growth factor (VEGF)-targeting therapy was performed for metastases in all 50 patients. They were treated with sunitinib according to a 4 weeks on/2 weeks off schedule (starting dose: 37.5 or 50 mg/day) or pazopanib (starting dose: 600 or 800 mg/day). Patients continued first-line therapy unless there was tumor progression, lack of response without progression, or intolerance. Subsequently, patients were given second-line VEGF-targeting therapy with axitinib (recommended starting dose: 10 mg/day). The effect of therapy was assessed according to the Response Evaluation Criteria in Solid Tumors (RECIST).

### Analysis of DNA samples

Before DNA analysis was performed, all patients gave written consent to analysis of somatic DNA by signing a form that was approved by our institutional Committee on Human Rights in Research, but only 5 patients gave approval for analysis of germline DNA. The DNA content of each sample was quantified and its purity assessed with a NanoDrop ND-1000 spectrophotometer (Labtech) [19]. This study was conducted according to the tenets of the Declaration of Helsinki and was approved by the ethical review board of Dokkyo Medical University Hospital.

### Next-generation sequencing

Tumor tissue samples of the 50 patients were used to investigate mutations of the Nrf2, Keap1, von Hippel-Lindau (VHL), and fumarate hydratase (FH) genes. We performed targeted next-generation sequencing of the coding exons and intron flanking regions of these four genes [19], using customized primers designed with Ampliseq Designer (Life Technologies). Construction of a library and sequencing were performed with an Ion AmpliSeq Library Kit 2.0, Ion PGM IC 200 kit, and Ion PGM (Life Technologies) according to the directions of the manufacturer. Raw data from each sequencing reaction were analyzed with Torrent Suite version 4.2.1.

### Real-time PCR

Genotyping of the rs6721961 SNP in the Nrf2 promoter region was performed by the real-time polymerase chain reaction with confronting two-pair primers (PCR-CTPP) [20].

### Immunohistochemical analysis

Immunohistochemical staining of tumor specimens from the 50 patients was done with an anti-Nrf2 monoclonal antibody (Abcam, # ab-62,352, Cambridge, UK) [19]. The tumors were divided into a low Nrf2 expression group (in which many tumor cells were weakly to moderately positive for anti-Nrf2 antibody and < 30% of all tumor cells were positive) and a high Nrf2 expression group (in which many tumor cells were moderately to strongly positive for anti-Nrf2 antibody and > 30% of all tumor cells were positive).

### Statistical analysis

Pearson’s χ^2^ test for contingency tables was employed to assess the association between Nrf2 polymorphism and Nrf2 protein expression, as well as the relation between the response to VEGF-targeting therapy and Nrf2 polymorphism or Nrf2 protein expression.

The Kaplan-Meier method was employed for estimation of cause-specific survival and the significance of differences in survival was examined by the log-rank test. Multivariate Cox proportional hazards analysis was performed to determine the influence of Nrf2 polymorphism, Nrf2 protein expression, histological grade, and lymph node metastasis on survival. In all analyses, *P* <  0.05 was accepted as indicating statistical significance. Analyses were performed with commercial software.

## Results

### Outcome of next-generation sequencing

According to targeted next-generation sequencing of primary tumor tissue samples, 5 out of 50 patients had SNPs of the Nrf2 gene associated with amino acid sequence variants. In addition, there were SNPs of Keap1 in 11 patients and SNPs of VHL in 35 patients, but no SNPs of FH were detected. There was no relationship between Nrf2 or Keap1 mutations and the histological grade, pT stage, or pN stage (Table 1).

**Table 1:** Relationship between molecular profiles and the pathologic factors

	Grade 1,2/3,4	*p* value	pT1,2/3,4	*p* value	pN0/1,2	*p* value
	(*n* = 14/36)		(*n* = 13/37)		(*n* = 32/18)	
Mutation (+) of Nrf2 (*n* = 5)	1 / 4	0.7471	0 / 5	0.1624	3 / 2	0.9687
No mutation of Nrf2 (*n* = 45)	13 / 32		13 / 32		29 / 16	
C/C at rs6721961 (*n* = 30)	8 / 22	0.7975	8 / 22	0.8953	19 / 11	0.4915
C/A or A/A at rs6721961 (*n* = 20)	6 / 14		5 / 15		13 / 7	
Nrf2 Low (*n* = 26)	8 / 18	0.6499	10 / 16	0.0365	19 / 7	0.1988
Nrf2 High (*n* = 24)	6 / 18		3 / 21		13 / 11	
Mutation (+) of Keap1 (*n* = 11)	1 / 10	0.1477	1 / 10	0.1477	6 / 5	0.2928
No mutation of Keap1 (*n* = 39)	13 / 36		12 / 27		26 / 13	

### Findings on molecular genetic analysis

**Fig. 1:**
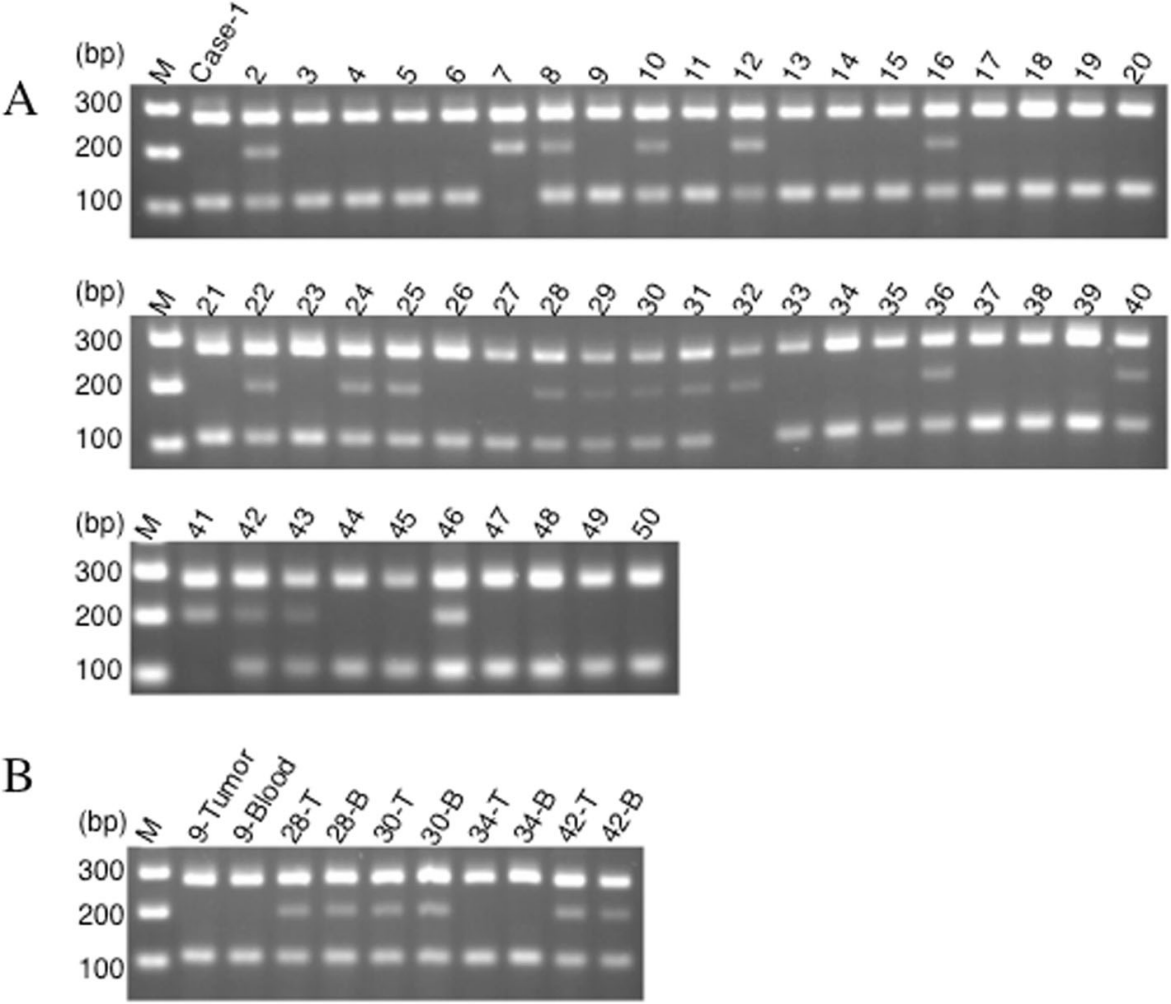
Expression of Nrf2 and genotyping of rs6721961 SNP. **a**: Gel showing the genotype for rs6721961 SNP of the *Nrf2* gene. C/C genotype (282, 113 bp), C/A genotype (282, 205, 113 bp), and A/A genotype (282, 205 bp). **b**: In five patients (case-9, 28, 30, 34, and 42), SNPs for rs6721961 examined from tumor (T) and blood (B) were identical

PCR-CTPP was performed to examine the rs6721961 SNP in the Nrf2 promoter region (Fig. 1a). When all 50 tumor samples were investigated, the three genotypes of this SNP showed the following frequencies: 60% (30 patients) for C/C, 34% (17 patients) for C/A, and 6% (3 patients) for A/A. Interestingly, the rs6721961 SNP was identical between germline and somatic DNA in 5 patients who consented to germline DNA analysis (Fig. 1b). This SNP showed no relationship with histological grade, pT stage, or pN stage (Table 1).

### Results of immunohistochmical analysis

**Fig. 2:**
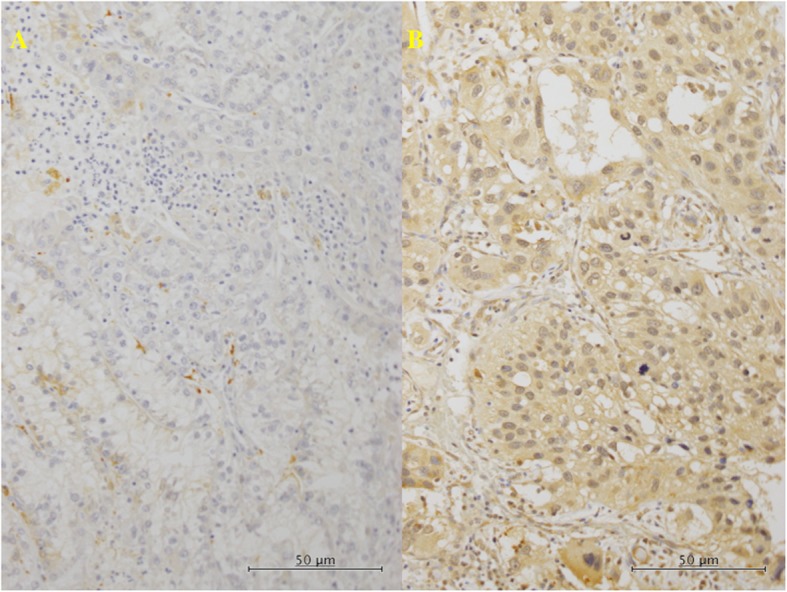
Immunohistochemistry in the primary tumor tissues for Nrf2. **a** (× 200): In the tumors with C/C genotype for rs6721961 SNP of the Nrf2, lower histological grade, some tumor cells showed weak reaction for anti-Nrf2 antibody (lower expression). **b** (× 200): In the tumors with C/A genotype, higher histological grade, much of the tumor cells showed moderate to strong brown staining (higher expression)

We divided the tumors into two groups, a low expression group (weak to moderate positivity) and a high expression group (moderate to strong positivity), depending on the level of positivity for anti-Nrf2 antibody (Fig. 2).

Tumors with Nrf2 gene mutations showed increased expression of Nrf2 protein, and the C/A and A/A genotypes of rs6721961 were significantly associated with elevated Nrf2 protein expression (*p* <  0.0001, Table 2). In contrast, Keap1 mutation showed no relation with Nrf2 protein expression (Table 2) or with localization of Nrf2. In addition, we found no association of Keap1 gene mutation with the level of Nrf2 expression after dividing the patients into a group with the C/C genotype of rs6721961 and a group with the C/A or A/A genotypes. Elevation of Nrf2 protein expression was associated with the tumor pT stage, but was not associated with the histological grade or pN stage (Table 1).

**Table 2:** Relationship between SNPs at rs6721961 and Nrf2 expression (*n* = 50)

	C/C at rs6721961 (*n* = 30)	C/A or A/A at rs6721961 (*n* = 20)	*p* value
Mutation (+) of Nrf2 (*n* = 5)	0	5	0.0543
No mutation of Nrf2 (*n* = 45)	30	15	
	Low in Nrf2 (*n* = 26)	High in Nrf2 (*n* = 24)	*p* value
Mutation (+) of Nrf2 (*n* = 5)	0	5	0.0184
No mutation of Nrf2 (*n* = 45)	26	19	
	Low in Nrf2 (*n* = 26)	High in Nrf2 (*n* = 24)	*p* value
C/C at rs6721961 (*n* = 30)	22	8	0.0005
C/A or A/A at rs6721961 (*n* = 20)	4	16	
	Mutation (+) of Nrf2 (*n* = 5)	No mutation of Nrf2 (*n* = 45)	*p* value
Mutation (+) of Keap1 (*n* = 11)	2	9	0.3057
No mutation of Keap12 (*n* = 39)	3	36	
	C/C at rs6721961 (*n* = 30)	C/A or A/A at rs6721961 (*n* = 20)	*p* value
Mutation (+) of Keap1 (*n* = 11)	7	4	0.7804
No mutation of Keap12 (*n* = 39)	23	16	
	Low in Nrf2 (*n* = 26)	High in Nrf2 (*n* = 24)	*p* value
Mutation (+) of Keap1 (*n* = 11)	4	7	0.3057
No mutation of Keap12 (*n* = 39)	22	17	

### Prognostic influence of Nrf2

When the primary tumor possessed Nrf2 gene mutations, the C/A or A/A genotypes of rs6721961, or elevated Nrf2 protein expression, metastatic lesions demonstrated a worse response to VEGF-targeting therapy (*p* = 0.0142, *p* = 0.0018, and *p* < 0.0001, respectively) (Table 3).

**Table 3:** Relationship between molecules and treatment response in metastatic lesions (*n* = 50)

	CR/PR/SD > 12w* (*n* = 26)	SD < 12w/PD* (*n* = 24)	*p* value
Mutation of Nrf2			
(+) (n = 5)	0	5	0.0142
(−) (n = 45)	26	19	
SNPs at rs6721961			
C/C (n = 30)	21	9	0.0018
C/A or A/A (n = 20)	5	15	
Nrf2 expresion			
Low (n = 26)	21	5	< 0.0001
High (n = 24)	5	19	
Mutation of Keap1			
(+) (n = 11)	6	5	0.8783
(−) (n = 39)	20	19	

CR/PR/SD > 12w* Complete response, partial response, or stable disease for > 12 weeks

SD < 12w/PD* Stable disease for < 12 weeks or progressive disease

**Fig. 3:**
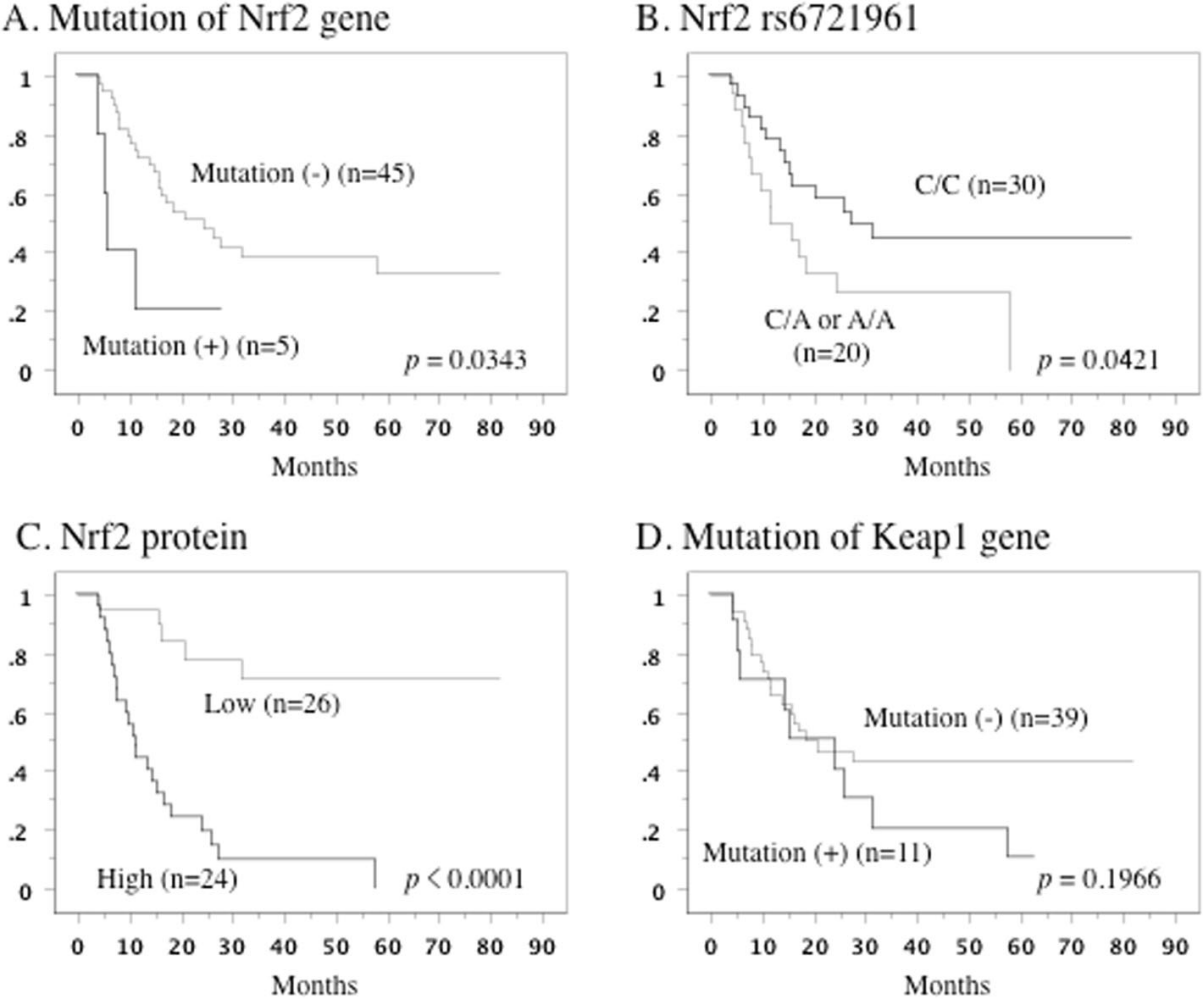
Overall survival curve in all patients. **a**: The patients who had tumors with Nrf2 gene mutation (+) showed shorter overall survival. **b**: The patients who had tumors with C/A or A/A genotype showed unfavorable overall survival. **c**: The patients with higher Nrf2 expression in the primary tumor showed worse overall survival. **d**: The Keap1 gene mutation was not associated with overall survival

When primary tumors had Nrf2 gene mutations and the C/A or A/A genotypes of rs6721961, Kaplan-Meier analysis showed that survival was shorter than if tumors had the C/C genotype (*p* = 0.0343 and *p* = 0.0421, respectively, Fig. 3a,b). In addition, overall survival was less favorable when the primary tumor showed higher Nrf2 expression (*p* < 0.0001, Fig. 3c). Keap1 gene mutations were also associated with shorter overall survival, but this association did not reach statistical significance (*p* = 0.1966, Fig. 3d), even after dividing the patients into a group with the C/C genotype of rs6721961 and a group with the C/A or A/A genotypes.

Although Nrf2 gene mutations were only detected in patients with the C/A or A/A genotypes of rs6721961, there were no differences of Nrf2 expression, the response to VEGF-targeting therapy, and overall survival between Nrf2 mutation (+) patients and Nrf2 mutation (−) patients with the C/A or A/A genotypes of RS 6721961.

According to univariate Cox proportional hazards analysis, Nrf2 gene mutations, the rs6721961 SNP, Nrf2 protein expression, histological grade, pT stage, and pN stage were all factors with a significant influence on overall survival, while Keap1 mutation was not (Table 4). Elevated expression of Nrf2 protein was confirmed to be an independent determinant of shorter survival by multivariate analysis (Table 4).

**Table 4:** Cox regression analysis for various potential prognostic factors in overall survival

Variable	Unfavorable/ favorable characteristics	No. of Patients	Univariate (U)			Multivariate (M)		
			Relative risk	95% confidential interval	*P* value	Relative risk	95% confidential interval	*P* value
Grade	4,3 / 2,1	14 / 36	2.579	1.425–4.667	0.0017	2.171	0.956–4.931	0.0639
pT	4,3 / 2,1	13 / 37	6.666	1.548–28.711	0.0109	4.222	0.745–23.922	0.1036
pN	2,1 / 0	18 / 32	2.365	1.095–5.110	0.0285	1.366	0.732–1.293	0.5042
Mutation of Nrf2	(+) / (−)	5 / 45	3.015	1.030–8.824	0.0440	2.791	0.610–12.768	0.1859
SNPs of rs6721961	CA, AA / CC	20 / 30	2.139	1.010–4.530	0.0469	1.167	0.857–1.351	0.7341
Expression of Nrf2	high / low	24 / 26	8.485	3.102–23.210	< 0.0001	6.435	1.964–21.087	0.0021
Mutation of Keap1	(+) / (−)	11 / 39	1.679	0.758–3.721	0.2016	1.122	0.891–1.347	0.8107

## Discussion

While Nrf2 protects cells against redox-mediated injury and carcinogenesis, it is also involved in oncogenic pathways. Constitutive activation of Nrf2 may occur in various human cancers and seems to be associated with tumor progression and a poor prognosis. Thus, Nrf2 can show either host-protective or tumor-promoting effects [1, 5].

It was reported that Nrf2 gene mutations leading to modification of the Nrf2 protein residues that interact with Keap1 cause activation of the cap’n’collar (CNC) – basic leucine zipper protein (bZIP) transcription factor [6]. Functional Keap1 mutations have been detected in various cancers, and these mutations lead to upregulation of Nrf2 / ARE gene transcription [1, 2]. In the present study, we used targeted next-generation sequencing for mutation analysis of primary tumor tissues from 50 patients metastatic RCC, and we detected Nrf2 gene mutation in 5 patients, Keap1 gene mutation in 11 patients, and VHL gene mutation in 35 patients. Abnormal VHL-mediated proteolysis is frequent in ccRCC, mainly due to biallelic inactivation of VHL resulting from allelic deletion or loss of heterozygosity together with gene mutation or promoter hypermethylation [21]. In the present study, we identified somatic VHL gene mutation in 35 of 50 tumors (70%). Sato et al. recently investigated copy numbers and/or methylation in over 100 ccRCC patients, using whole-genome and/or whole-exome sequencing, RNA sequencing, and microarray analysis, and they identified a new Keap1-Nrf2 pathway mutation along with VHL mutation [22]. They confirmed mutual exclusivity of Keap1 and Nrf2 mutation in 6.6% of their patients. In this study, we identified Nrf2 mutations (*n* = 3), Keap1 mutations (*n* = 9), and mutations of both genes (*n* = 2) in the primary tumors of 14/50 patients with metastatic ccRCC (28%). Our patients had metastatic ccRCC with poor histology, local invasion, or regional lymph node involvement, but Sato et al. did not report the clinicopathological characteristics of their ccRCC patients, including the histological grade or stage. Therefore, we cannot compare clinicopathological characteristics between the two studies. However, it is possible that Keap1 and/or Nrf2 mutations might have a more important role in RCC than has been recognized up to now, particularly in patients with biologically aggressive tumors.

It is still unclear how Nrf2 is activated in ccRCC, but its activation may be dependent on dysregulation of tumor suppressor genes and oncogenic pathways [1, 2]. It was reported that Nrf2 mutation results in a sustained activation of Nrf2in sporadic papillary RCC (pRCC) [23]. Keap1 is an adaptor protein that facilitates ubiquitination and subsequent degradation of Nrf2, which means that Keap1 mutation can lead to sustained Nrf2 activation [1–3]. In the present study, we found that Nrf2 gene mutation was associated with elevated Nrf2 protein expression, while Keap1 mutation was not. However, all of our patients had metastasis (M1) and many of them had poorly differentiated tumors (grade 3/4), invasive disease (pT3/4) or lymph node involvement (pN1–2) (Table 2). In our preliminary study, we did not detect Nrf2 or Keap1 mutation in patients with higher differentiated (grade 1/2), noninvasive (pT1), or non-metastatic (M0) tumors (data not shown). Therefore, although the mechanism underlying the association of the Nrf2 gene mutations with progression of ccRCC is unclear, it seems likely that mutations of both genes stabilize Nrf2 by disrupting its binding to Keap1 and that sustained activation of Nrf2 could be a prominent feature of ccRCC. On the other hand, inactivating mutation of FH (an enzyme involved in the mitochondrial tricarboxylic acid cycle) causes Nrf2-dependent activation of antioxidant pathways in patients with inherited type 2 pRCC (pRCC2) or hereditary leiomyomatosis and renal cell carcinoma (HLRCC). In patients with these tumors, succination of Keap1 results in stabilization of Nrf2 and induction of stress-response genes to promote the survival of FH-deficient cells, and Nrf2 influences the intracellular fumarate level [24, 25]. However, previous studies have not identified FH mutations in ccRCC cell lines or primary ccRCC specimens [26, 27], and we did not detect any somatic FH gene mutations in 50 ccRCC specimens in this study. Therefore, unlike pRCC, other signaling networks might interact with the Nrf2 pathway during progression of ccRCC.

Suzuki et al. recently reported that the rs6721961 SNP in the ARE-like sequence of the human Nrf2 promoter was associated with an elevated risk of lung cancer, especially among smokers [10]. They demonstrated significant reduction of the Nrf2 mRNA level by approximately 40% in A/A homozygotes for the rs6721961 SNP compared with C/A heterozygotes or C/C homozygotes, and the risk of lung cancer was only increased for A/A homozygotes. Based on these results, homozygous substitution of A for C at rs6721961 significantly decreases Nrf2 mRNA expression, and Nrf2 normally protects against carcinogenesis in humans. In contrast to Suzuki et al., we studied primary ccRCC and we found that the genotype frequencies of the rs6721961 SNP were 60% for C/C, 34% for C/A, and 6% for A/A. The C/A and A/A genotypes were both significantly associated with increased Nrf2 protein expression (*p* < 0.0001), and metastases showed a worse response to VEGF-targeting therapy with shorter overall survival if the primary tumor possessed the C/A or A/A genotype or showed elevation of Nrf2 protein expression. Although we did not examine Nrf2 mRNA expression in the ccRCC specimens, these observations suggest that tumor-specific and/or organ-specific variation of Nrf2 transcription and expression mediated via rs6721961 or other SNPs could have a role in various human diseases.

Oxidative phosphorylation is impaired in RCC, with a resulting metabolic shift to aerobic glycolysis [28]. According to Mitsuishi et al., elevation of Nrf2 expression redirects glucose and glutamine to anabolic pathways and constitutive activation of Nrf2 increases metabolic activity to support cell proliferation, suggesting that Nrf2 activation is associated with metabolic reprogramming [4]. We previously found increased Nrf2 expression and aerobic glycolysis in HLRCC tumor cells with FH mutation [19]. Therefore, Nrf2 pathway activation may promote aerobic glycolysis in RCC.

There were several limitations of present study had, including its retrospective design, relatively small study population, and short follow-up period that did not allow definite conclusions regarding the influence of Nrf2 on the prognosis of ccRCC. We performed a retrospective investigation of the relationship between Nrf2 and clinicopathological features in patients who were given first-line or second-line VEGF-targeting therapy. However, various factors influence the treatment recommended for patients, which might also have a significant effect on the response and overall survival. Also, we studied Nrf2 gene mutations, the rs6721961 SNP, and Nrf2 protein expression in the surgically resected primary tumors of patients with metastatic ccRCC. It is unlikely that the biological characteristics of primary and metastatic tumors would be identical and treatment strategies probably should be reconsidered by taking into account intra-patient tumor heterogeneity [29]. Therefore, we should not only study Nrf2 gene mutations, the rs6721961 SNP, and Nrf2 protein expression in early ccRCC, but we should also examine metastatic lesions in order to elucidate the role of Nrf2 in tumor progression. Accordingly, the present findings require confirmation by further studies, preferably prospective clinical trials on a larger scale. Sequencing of the human genome and development of high throughput methods have made rapid analysis of a large number of SNPs possible. Each patient’s genetic profile influences the response to therapy. Although we only tested 5 tumor samples in the present study, we found that the rs6721961 SNP was identical between germline and somatic DNA in all 5 patients. If this SNP is completely identical between germline and tumor DNA, examination of the germline DNA might be useful for monitoring tumor behavior and responses. Moreover, more detailed investigation of Nrf2 gene mutations is required, by sequencing the coding exons and intron flanking regions in peripheral blood leukocytes and tumor samples acquired from a larger patient population. Further investigation of the mechanisms underlying the dual role of Nrf2 in both suppressing and promoting growth of ccRCC is needed, in order to provide a theoretical basis for novel mechanisms of cancer progression and resistance, and to find new molecular targets to enhance the sensitivity of this tumor to treatment.

## Conclusions

When primary RCC displayed Nrf2 gene mutation and the C/A or A/A genotype of rs6721961 at the Nrf2 promoter region, expression of Nrf2 was elevated and metastases showed a worse response to sequential vascular endothelial growth factor-targeting therapy, resulting in unfavorable survival. These findings suggest that Nrf2 signaling is important in the progression of RCC.

## Data Availability

The datasets generated and analyzed during the current study are available from the corresponding author on reasonable request.

## Abbreviations

ARE: Antioxidant response element
bZIP: basic leucine zipper protein
ccRCC: clear cell renal cell carcinoma
CNC: Cap'’n'’collar
CT: Computed tomography
FH: Fumarate hydratase
HLRCC: Hereditary leiomyomatosis and renal cell carcinoma
Keap1: Kelch-like ECH-associated protein 1
Krebs cycle: Mitochondrial tricarboxylic acid cycle
LOH: Heterozygosity
LVI: Lymphovascular invasion
MRI: Magnetic resonance imaging
Nrf2: Nuclear factor erythroid-2-related factor 2
PCR-CTPP: Real-time polymerase chain reaction with confronting two-pair primers
pRCC: papillary renal cell carcinoma
pT: pathological Tumor
RCC: Renal cell carcinoma
RECIST: Response Evaluation Criteria in Solid Tumors;
ROS: Reactive oxygen species
S.D.: Standard deviation
SNPs: Single nucleotide polymorphisms
TNM: The TNM classification of Malignant Tumors (T: tumor, N: lymph node, M: metastasis)
VEGF: Vascular endothelial growth factor
VHL: Von Hippel-Lindau
